# LB750. Safety and Immunogenicity of a Bivalent Omicron-Containing Booster Vaccine against COVID-19

**DOI:** 10.1093/ofid/ofac492.1873

**Published:** 2022-12-15

**Authors:** Spyros Chalkias, Charles Harper, Keith Vrbicky, Stephen R Walsh, Brandon Essink, Adam Brosz, Nichole McGhee, Joanne Tomassini, Xing Chen, Ying Chang, Andrea Sutherland, David Montefiori, Bethany Girard, Darin Edwards, Jing Feng, Honghong Zhou, Lindsey R Baden, Jacqueline Miller, Rituparna Das

**Affiliations:** Moderna, Inc., Cambridge, MA; Meridian Clinical Research, Norfolk, Nebraska; Meridian Clinical Research, Norfolk, Nebraska; Brigham and Women's Hospital, Boston, Boston, Massachusetts; Meridian Clinical Research, Norfolk, Nebraska; Meridian Clinical Research, Norfolk, Nebraska; Moderna, Inc., Cambridge, MA; Moderna, Inc., Cambridge, MA; Moderna, Inc., Cambridge, MA; Moderna, Inc., Cambridge, MA; Moderna, Inc., Cambridge, MA; Department of Surgery and Duke Human Vaccine Institute, Durham, North Carolina; Moderna, Inc., Cambridge, MA; Moderna, Inc., Cambridge, MA; Moderna, Inc., Cambridge, MA; Moderna, Inc., Cambridge, MA; Brigham and Women's Hospital, Boston, Massachusetts; Moderna, Inc., Cambridge, MA; Moderna, Inc., Cambridge, MA

## Abstract

**Background:**

Vaccination strategies that provide enhanced immunity against severe acute respiratory syndrome coronavirus 2 (SARS-CoV-2) variants are needed. We evaluated the safety and immunogenicity of a bivalent omicron containing vaccine, mRNA-1273.214 (50 µg), administered as a second booster dose in adult participants.

**Methods:**

In this ongoing phase 2/3 trial, 50 µg of the bivalent vaccine mRNA-1273.214 (25 µg each ancestral Wuhan-Hu-1 and omicron BA.1 spike mRNAs) or 50 µg of the authorized mRNA-1273 were administered as second boosters in adults who previously received a 2 dose (100 µg) primary series and a first booster (50 µg) dose of mRNA-1273 (≥ 3 months prior). Primary objectives were safety and reactogenicity and immunogenicity 28 days post-booster dose.

**Results:**

In participants with no prior SARS-CoV-2 infection who received booster doses of mRNA-1273.214 (n=334) or mRNA-1273 (n=260), neutralizing antibody (nAb) geometric mean titers (GMTs [95% confidence interval (CI)]) against omicron BA.1 were 2372.4 (2070.6−2718.2) and 1473.5 (1270.8−1708.4), respectively. The model-based GMT ratio (GMR [97.5% CI]) of mRNA-1273.214 compared to mRNA-1273 was 1.75 (1.49−2.04), meeting the pre-specified superiority criterion against omicron BA.1. The pre-specified criterion for non-inferiority against the ancestral SARS-CoV-2 strain was also met. Additionally, mRNA-1273.214 elicited higher GMTs (727.4 [632.8−836.1]) than mRNA-1273 (492.1 [431.1−561.9]) against omicron subvariants BA.4/BA.5 [GMR (95% CI) 1.69 [1.51−1.90])]. Binding antibody responses against alpha, beta, gamma, delta, and omicron were numerically higher in the mRNA-1273.214 group compared to mRNA-1273. mRNA-1273.214 GMTs were consistently higher across age (18-< 65 and ≥ 65 years) and pre-booster SARS-CoV-2 infection subgroups (**Figure**). Safety and reactogenicity were similar for both vaccine groups.

**Figure:**
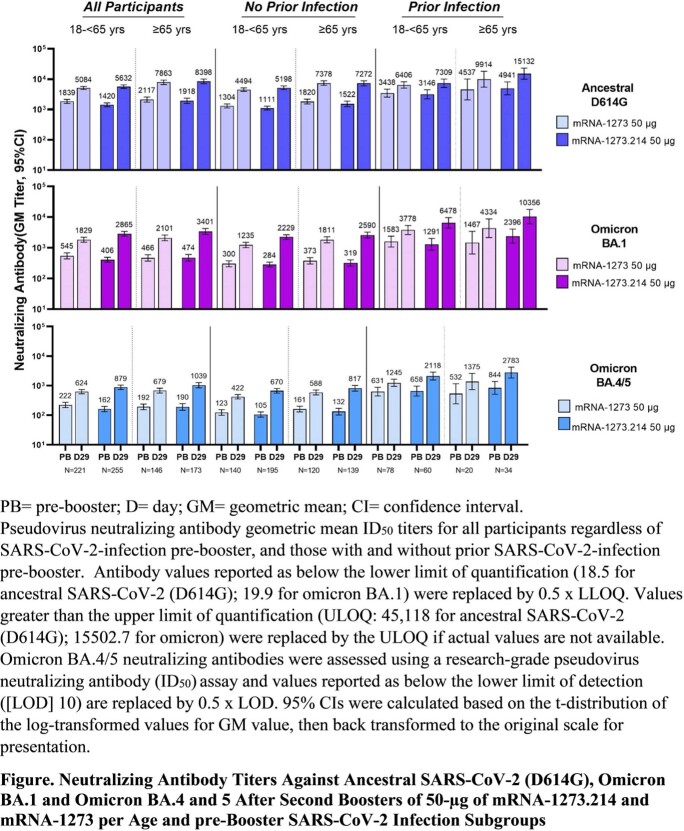

**Conclusion:**

The bivalent omicron containing mRNA-1273.214 elicited superior nAb responses against omicron 28 days post-immunization compared to mRNA-1273 regardless of age and prior SARS-CoV-2 infection; no new safety concerns were identified.

**Disclosures:**

**Spyros Chalkias, MD**, Moderna, Inc.: Salary|Moderna, Inc.: Stocks/Bonds **Stephen R. Walsh, MD**, Janssen Vaccines: Grant/Research Support|Moderna, Inc.: Grant/Research Support|NIAID/NIH: Grant/Research Support|Sanofi Pasteur: Grant/Research Support **Nichole McGhee, B.S.**, Moderna, Inc.: Salary|Moderna, Inc.: Stocks/Bonds **Joanne Tomassini, Ph.D.**, Moderna, Inc.: Advisor/Consultant **Xing Chen, Sc.D.**, Moderna, Inc.: Salary|Moderna, Inc.: Stocks/Bonds **Ying Chang, M.S.**, Moderna, Inc.: Salary|Moderna, Inc.: Stocks/Bonds **Andrea Sutherland, M.D., MPH**, Moderna, Inc.: Salary|Moderna, Inc.: Stocks/Bonds **David Montefiori, Ph.D.**, Moderna, Inc.: Grant/Research Support **Bethany Girard, Ph.D.**, Moderna, Inc.: Salary|Moderna, Inc.: Stocks/Bonds **Darin Edwards, Ph.D.**, Moderna, Inc.: Salary|Moderna, Inc.: Stocks/Bonds **Jing Feng, M.S.**, Moderna, Inc.: Salary|Moderna, Inc.: Stocks/Bonds **Honghong Zhou, Ph.D.**, Moderna, Inc.: Salary|Moderna, Inc.: Stocks/Bonds **Lindsey R. Baden, MD**, Moderna, Inc.: Grant/Research Support|NIAID: Grant/Research Support **Jacqueline Miller, MD**, Moderna, Inc.: Salary|Moderna, Inc.: Stocks/Bonds **Rituparna Das, M.D.**, Moderna, Inc.: Salary|Moderna, Inc.: Stocks/Bonds.

